# Supplementary material for: Griseofulvin Inhibits Root Growth by Targeting Microtubule-Associated Proteins Rather Tubulins in Arabidopsis
Source: Int J Mol Sci. 2023 May 12;24(10):8692. doi: 10.3390/ijms24108692 (PMC10217847; doi:10.3390/ijms24108692)
Supplement: Supplementary file 1 [file ijms-24-08692-s001.zip › Supplementary Material/Supplementary Material/Figure S1.pdf]

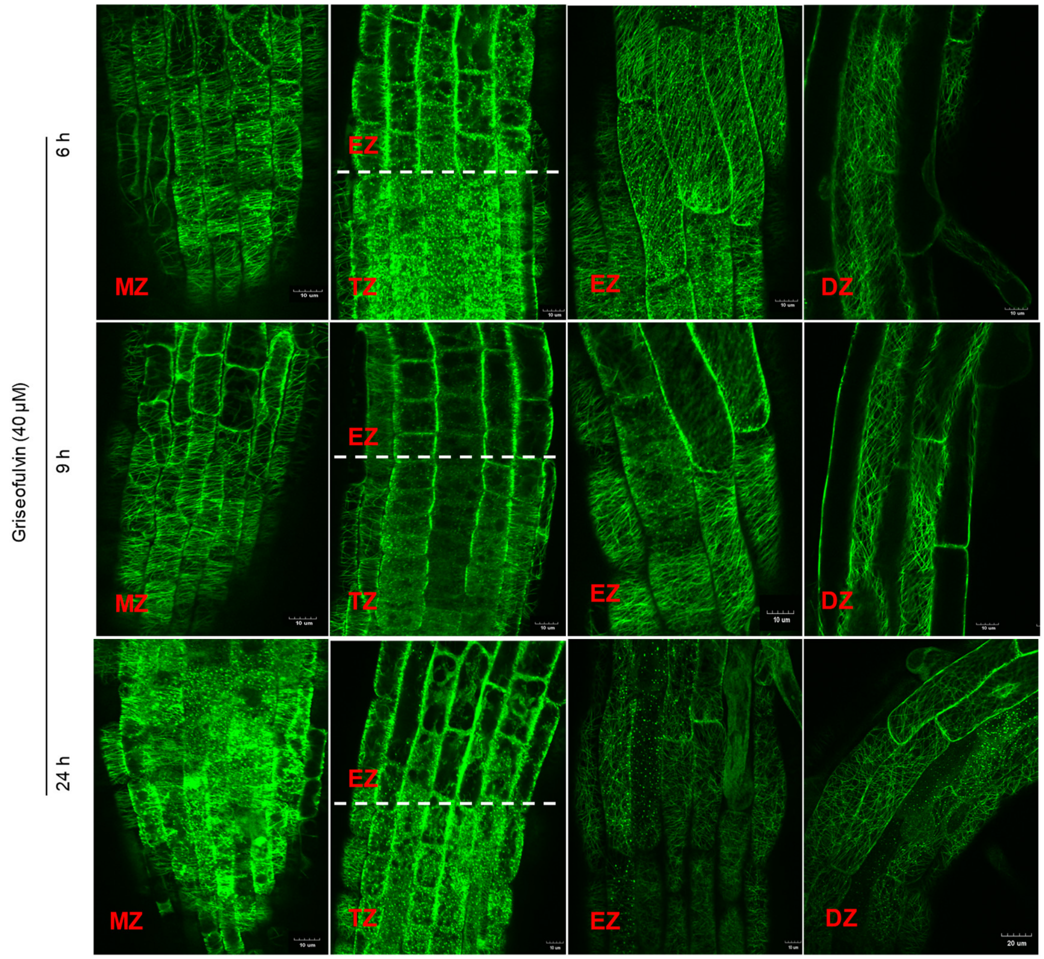

Figure S1. Effect of 40 μM griseofulvin on microtubule dynamics of root tip cells in *Arabidopsis*.

Five-day-old MBD-GFP seedlings growth on the conventional 1/2 MS medium were transferred onto 1/2 MS medium 40 μM of griseofulvin and then incubated for 6, 9 and 24 h. Microtubule dynamics of different zones of root tips after griseofulvin incubation were shown. Results represent three independent biological replicates. Scale bar: 10 μm or 20 μm.
