# Supplementary material for: Griseofulvin Inhibits Root Growth by Targeting Microtubule-Associated Proteins Rather Tubulins in Arabidopsis
Source: Int J Mol Sci. 2023 May 12;24(10):8692. doi: 10.3390/ijms24108692 (PMC10217847; doi:10.3390/ijms24108692)
Supplement: Supplementary file 1 [file ijms-24-08692-s001.zip › Supplementary Material/Supplementary Material/Figure S2.pdf]

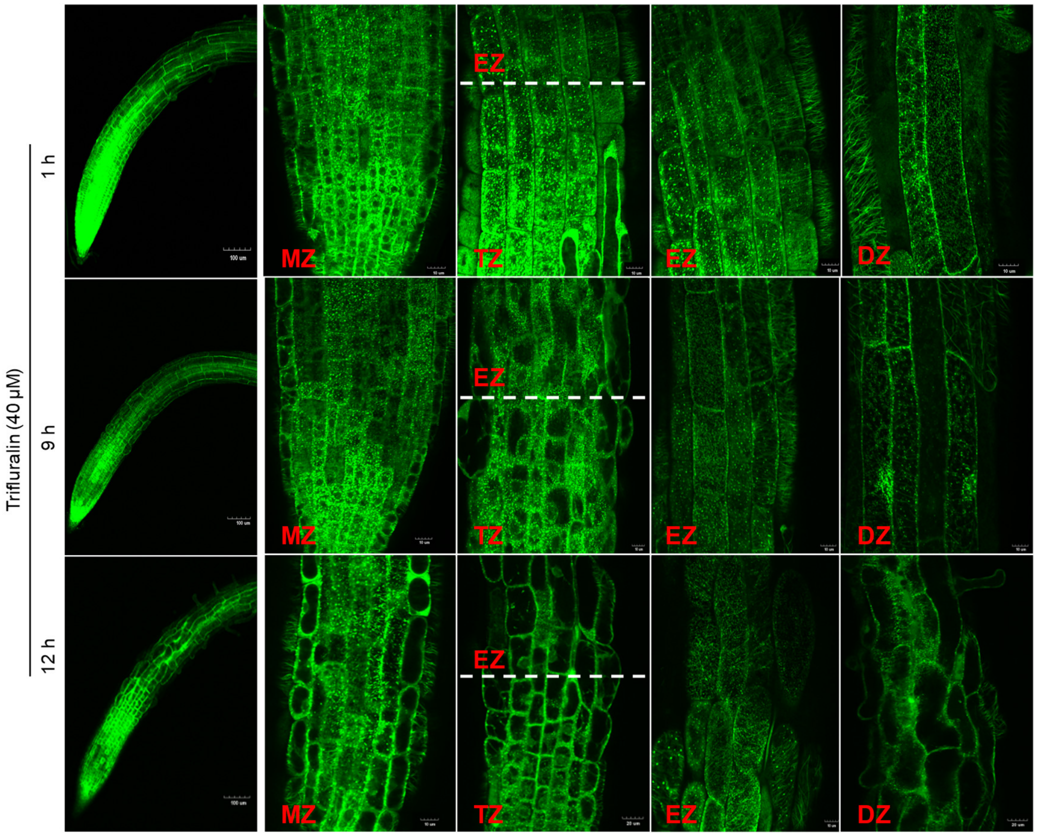

Figure S2. Effect of 40 μM trifluralin on microtubule dynamics of root tip cells in *Arabidopsis*.

Five-day-old MBD-GFP seedlings growth on the conventional 1/2 MS medium were transferred onto 1/2 MS medium 40 μM of trifluralin and then incubated for 1, 9 and 12 h. The overall morphology (left, scale bar: 100 μm) and microtubule dynamics (right, scale bar: 10 μm or 20 μm) of different zones of root tips after trifluralin incubation were shown. Results represent three independent biological replicates.
