# Supplementary material for: Griseofulvin Inhibits Root Growth by Targeting Microtubule-Associated Proteins Rather Tubulins in Arabidopsis
Source: Int J Mol Sci. 2023 May 12;24(10):8692. doi: 10.3390/ijms24108692 (PMC10217847; doi:10.3390/ijms24108692)
Supplement: Supplementary file 1 [file ijms-24-08692-s001.zip › Supplementary Material/Supplementary Material/Figure S3.pdf]

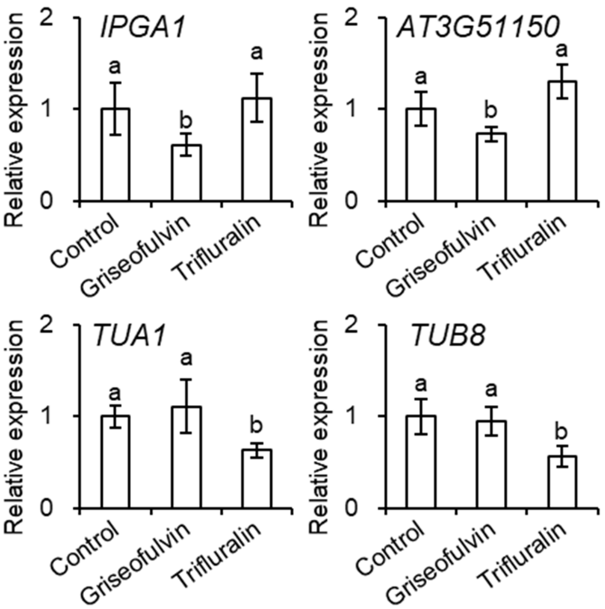

Figure S3. The expression levels of selected plant MAPs and tubulin-related genes including *IPGA1*, *AT3G51150*, *TUA1* and *TUB8* in Col-0 seedlings inoculated with griseofulvin or trifluralin were measured by quantitative PCR (qRT-PCR). Gene expression levels were normalized to *ACTIN2*. Data are mean ± SE of three independent biological replicates. The different small letters above error bars indicate significant difference at 0.05 level.
